# Supplementary material for: When to wake up? The optimal waking-up strategies for starvation-induced persistence
Source: PLoS Comput Biol. 2021 Feb 11;17(2):e1008655. doi: 10.1371/journal.pcbi.1008655 (PMC7904209; doi:10.1371/journal.pcbi.1008655)
Supplement: Attachment — Submitted filename: Reply.docx [file pcbi.1008655.s004.docx]

Dear Editor,

Thank you for your consideration of our manuscript “When to wake up? The optimal waking-up strategies for starvation-induced persistence‘’ submitted to *PLOS Computational Biology.* Hereby we would like to resubmit the revised manuscript.

We have updated the main manuscripts and added several paragraphs in the S1 text. All the major changes are highlighted in red in the manuscript (according to the request, the changes in the S1 text are not highlighted)

We believe that the revised manuscript meets all the requests by the referee. Thank you again for your kind consideration.

Sincerely yours,

Yusuke Himeoka and Namiko Mitarai

================

Reply to Reviewer #1

================

Thank you very much for the positive evaluation of the manuscript. We answer your comments below.

*Comment: The prediction of the locally optimal strategies in Fig. 3 is very interesting. Are these locally optimal strategies stable in the experiment? If so, is there any published evidence to support this?*

Reply: Naively speaking, the stability of the locally optimal solution depends on the probability of having a mutation that overcomes the local barrier in the fitness, but it is hard to evaluate the likelihood of such mutation, especially because the lagtime is affected by various physiology controlling genes. As far as we know, there is no published experimental evidence about the locally optimal strategy. It would be exciting if this work inspires a future experimental investigation in this direction.

*Comment: In Fig. 3cd, why are the curves of the λa, λb only shown in some range of the Antibiotics Application Time (T)?*

Reply: We did not plot the optimal lag time value if the corresponding population has zero fraction in the whole population, because any value is allowed. This is stated in the figure caption of Fig.3.

*Comment: The prediction that the optimal strategy changes to bet some fraction of the population to zero-lab time for larger T is very interesting. Can the authors do some simple simulation to further demonstrate the prediction?*

Reply: We are glad that the reviewer found this finding interesting. According to the comment, we have tried to perform a stochastic simulation of mutation and selection to see if the system actually evolves to this found optimal solution. The straightforward way is to do the same simulation as Fig.1 (c) and (d) with (λa,λb,x) as parameters of each type. However, the increased parameter space meant that the simulation is numerically challenging to perform within a reasonable time with the resource we currently have, and improving the algorithm will take a substantial amount of time. Therefore, we would like to leave this point as one of the future projects.

*Comment: The meaning of the bistable behavior in Fig.1 is not clearly described. Would the distribution of the lag time in one population show bimodal under this case? Is it related to the Bet-hedging case?*

Reply: In each round of the in-silico waking up experiment, the lag time distribution is single-peaked. However, the peak hops between the lower- and upper value of Fig.1D. If we average the lag time distribution for multiple rounds, it becomes a bimodal distribution. We would think this bistable behavior emerges because we allow the population to have only one phenotype. We added a sentence to describe this point to the revised manuscript.

================

Reply to Reviewer #2

================

Thank you very much for the thorough review. Below we answer your concerns.

Major concerns:

*1) In the section “comparison with sequential selection procedure”, the authors assume that the mutations are continuous and can occur only between closely related phenotypes, which is problematic and not realistic. I wonder what would happen if they considered a random switching case?*

Reply: In the original model, we assumed a mutation causes relatively small change in the lag time length. However, since the various mutations can affect lag time in different ways in complex mannar, some mutations can have significant impact on lag time as the reviewer pointed out. Therefore, we performed a new simulation with random switching, where any phenotype can change to any other phenotype in the same probability. The time average of the population-averaged lag time showed a continuous increase as expected, because the noise level increased dramatically. However, looking at the population-averaged lag time at each round, we found that it showed bistable-like behaviour. Most importantly, the selected phenotype had the expected optimal lag time. Overall,the feature of the dynamics itself is unchanged, but the time scale of the evolution is accelerated. The results are described in the supplemental section 3.

*2) In figure 2, the authors show that increasing both, the mean exposure-time and its standard deviation, increases the steepness of the transition in optimal lag-time in response to increasing probability of encountering stress. While the result for increasing mean exposure-time might be somewhat clear to me, I do not understand why the increase of standard deviation would have a similar effect. This needs to be explained better.*

Reply: This is because the chance of the exposure time (*T*) being long is higher if the standard deviation is increased for a fixed average. This means that the benefit of longer lag time becomes larger. We added one line to explain this.

Also, we noticed that the transition of the optimal lag time becomes discontinuous for large average or standard deviation. In the revised manuscript, we addressed this point and added an analytical calculation that shows that the optimal lag time can exhibit the discontinuous transition even if the lower bound of the support of q(T) is greater than 0.

*3) In the section “Multi-step sequential wakeup model”, the authors consider a scenario in which the cells go through several stages before waking up. Since the different stages have exactly the same behavior, and the transition into growth state occurs only from the final stage of the dormant states, I do not see how this model is different from the one dormant state model with different waking up time? The authors need to explain better what is different here.*

Reply: Even the transition rate from the ith to (i+1)th state is the same (and scaled by the number of states) and growth takes place only in the growing state, the waking-up time (the duration to pass the final dormancy state to the growing state) distribution is different; it follows the Erlang distribution. We scaled the rate λ by the number of steps so that the mean is the same for any M (total number of steps), but higher moments such as variance depends on M (larger M gives smaller variance). In the revised manuscript, we explicitly wrote down the Erlang distribution and noted the difference by having M>1.

*4) In the section “Bet-hedging” I failed to understand what is xL*?*

Reply: It is the locally optimal fraction (the fraction performs better than slightly less or more fraction (*xL*±ε*), but not globally optimal). We added lines for the explanation of xL* also in the caption of Fig.3 in the main manuscript.

*5) In the section “ General form of the optimal lag time distribution”, the authors assume a specific form of distribution, which seems to split the population into two fractions, one fraction (alpha) that has a lag time zero, and the rest have a distribution of lag times (s(l)). They claim that this is the optimal form and that they prove it in the supplementary info. I could not really see how they prove that. Are they considering in their calculations the possibility that alpha could be zero? I would actually like to see how the fitness change with the value of alpha. It seems to me that this assumption limits the possibilities for s(l), which then appear to follow the distribution of exposure times. This is not surprising, since it actually means that the cells should be more or less responding to their environment. I wonder if for alpha equal to zero, they would get s(l) that completely mimics the distribution of exposure times?*

Reply: Thanks to the reviewer's comment, we realized that our previous manuscript could have confused the readers about what has been proven and what has been deduced from it. First, as long as 0<=α<=1 (notice α=0 is included) and s(l) is an arbitrary function, it is always true that any function r(l) can be written as
r(l) = αδ(l) + (1-α)s(l)
because if r(l) has the delta function at the origin, one can set α to non-zero, and otherwise, set α=0 with defining s(l)=r(l). This division is always possible.

We have proven two facts; (1) minsupp(s) > 0 and (2) α continuously change with *p, γ*, and *q(T)*.

We first start with the first claim, minsupp(s) > 0. Here, *min* is minimum, and *supp* is the support of the function which is a region where the function has a non-zero value. Thus, minsupp(s) > 0 means that the function s(l) is exactly zero in a finite range around l=0. The reason why we divide *r* into two parts is to clarify this point. This argument holds for any value of α from zero to one. (Note that all the calculations in the proof are valid even if α=0 is substituted.)

While we dealt with α as if it is a parameter to show minsupp(s) > 0, it is not actually the parameter which can be freely set, but it is obtained by the optimization as a function of *p, γ*, and *q(T)*. The second statement claims that α=α(p,γ,q) is a continuous function of *p, γ*, and *q(T)*. This statement is necessary to state “*the existence of the gap between the origin and minsupp(s(l)) means that another peak of the optimal distribution never stems from the delta peak at the origin, but discontinuously appears as p, γ or q(T) changes*” in line 304-306 in the first version of the manuscript.

Since α is not a free parameter, but determined by the optimization, it is not easy to compute the optimal distribution under fixed α. However, we can easily compute the optimal distribution specifically under α=0 (compute the optimal distribution under the condition that the bin at origin is zero). Here, we denote this as k(l). Then, we can compare the fitness of the distribution function αδ(l) + (1-α)k(l) by changing α value. As shown in figure below, the distribution with α=0 has lower fitness value than that with non-zero α even though the function k(l) is not optimized for non-zero α.
(Rest of the setup is the same with that for Fig.5(a) in the main text.)

We like to emphasize that we assume nothing about s(l). We only assumed that s(l) is a probability distribution function. Also, the main point of the section is that r(l) does not completely mimic the distribution of exposure time even though we allowed r(l) to be any probability distribution function; this can be seen not only in that it has the delta-function at the origin but also the sharp cut-offs of the s(l). This is in a strong contrast with the type-II persister model.

We rewrote the corresponding paragraphs to clearly state what we assumed and proved. Also, we stressed α can be 0.

*6) Section “Comparison of the optimal lag time distribution and the results using the multi-step sequential wake-up model”. Again, it is not clear what the model is here and how is that different that changing the lag time distribution (see major concern 3).*

Reply: We believe that our reply to the major concern 3 answers this comment, too.

Minor concerns:

*1) Normally, starvation-induced persistence is a response to stress, which can occur in various stressful conditions albeit at different rates. While I think the authors have the right not to consider the possibility of transitioning back to dormant state when the bacteria experience antibiotics, I think that considering this possibility would have made their results more realistic for comparison with experiments.*

Reply: We here consider the situation where a lethal amount of bactericidal antibiotic is added to the environment, hence there is little chance to adapt to the stress, which we believe is a realistic scenario. However, we agree that in general stress sensing and adaptation is an interesting extension to consider. We have discussed this possibility in the discussion section (in line 521-531). It is however a significant extension of the present paper and we leave it for a future work.

*2) The results presented in the paper are not surprising given the assumptions made by the authors. The authors claim that this study can help in planning antibiotic treatments in order to over come this hurdle of bacteria surviving the treatment through persistence. However, I failed to see how this study contributes to that. In my opinion, it would have been more informative to investigate realistic situations like using real lag-time distributions and find what is the best strategy to kill them.*

Reply: If a finding is “surprising” or not is rather subjective, but considering the other comments from the reviewer, the “not surprising” judgement may be partly due to misunderstanding. Especially, as stated in reply to the major concern 5), we have proven several non-trivial features of the optimal lag time distribution, and it is not just mimicking the distribution of exposure time. Also, in Fig. 6 and related text, we have demonstrated how the fitness close to the optimal one can be realized in a specific wake-up dynamics (hence within limited lag time distribution function). This shows that it is likely for the obtained theoretical optimal has relevance in the real system, where the wake-up dynamics is limited by the possible molecular processes. We believe these are fairly non-trivial results.

At the same time, we agree that the current analysis is limited to an idealized setup, and there are various complications in real clinical applications. However, fully understanding what is expected in a simple, well-defined setup is necessary first step to understand more realistic and complex situations, since it is impossible to simulate all possible scenarios. This work is the first thorough theoretical analysis that focuses on the type I persistence, in contrast to the type II persistence where many theoretical works were done, where often the assumption for the growth condition is simplified as always nutrient rich. We also believe our framework can be used to simulate more complex scenarios. In the present manuscript, we extended the paragraph at the end of the discussion to state these considerations.

*3) What is minsupp()?*

Reply:
Please refer to the reply to major concern #5.

*4) Line 221: typo, “bed-hedging” instead of “bet-hedging”.*

*5) Line 293: typo, “as” should be “is”.*

*6) Line 320 – 321: typo, “… r and q is the vector of … function… element…” should be “… r and q are the vectors of … functions… elements…”.*

Reply:
Thank you very much for pointing out the typos. We collected them. About “as” and “is” (#5), “and” was not for connecting two sentences, but connecting two nouns “the antibiotics application itself” and “the duration”. The sentence might have been confusing. We rewrote the sentences.

================

Reply to Reviewer #3

================

*A theoretical understanding of how dormant cells interact with antibiotics is a worthwhile endeavor given the importance for understanding why antibiotic (or chemotherapy, etc) treatments fail. This effort examines a very specific circumstance where fresh nutrients (encouraging growth) and antibiotics are delivered simultaneously, but antibiotics are removed before fresh growth media is. It is hard to imagine how this scenario might play out in a patient. That doesn’t necessarily make the model less valuable, as one needs to make assumptions and see how those play out in the model, but it would be nice to deepen the discussion of what biological phenomena we really expect this model to predict.*

Reply: Thank you for the careful evaluation. The present setup, which is inspired by the experiment by Fridman et al. (2014), is indeed an idealized setup in the lab, and as it is unlikely to directly apply to more complex clinical situation.

One of the main achievement of the present work that has a wide impact is establishing the optimal strategy for the type-I (triggered) persistence in an idealized setup, and clarify the similarity and contrast to the type-II (spontaneous) persistence, where the theoretical analysis typically (implicitly or explicitly) always “nutrient rich” and the cells can grow exponentially when the “stress” is removed. Such analysis for type-II persistence were also quite simplified, but they significantly contributed to the field in fundamentally understanding the fitness of type-II persistence and hence how such phenotype can be evolved. We believe our analysis contributes similarly to the type-I persistence. This consideration is more thoroughly discussed in the manuscript.

Another significant achievement is establishing a solid framework that can be easily applied to the more realistic situations. One of the straightforward extensions of the present analysis, that can be closer to a more realistic scenario, could be considering two stresses - one is starvation, another is the antibiotic application, and let both of the stresses fluctuate. Our present analysis is the limiting case of such a situation, where the nutrient and antibiotic arrives at the same time. The disappearance of the antibiotic after sometime can be considered as the lifetime of the antibiotic or diluted below the minimal inhibitory concentration before the nutrient is fully consumed. One can of course consider different correlation (or lack of correlation) in the nutrients and the antibiotics in the environment. In general case, it will be also relevant to mix the type I and type II persistence, since if the antibiotic comes in long after the cells are in the nutrient rich condition and nutrient is still available, type II persister is likely more important. We believe that one of the important contributions of our present analysis to the field is to provide a solid modeling framework that can be extended to such situations.

In the updated manuscript, we strengthened these considerations at the end of the discussion.

Specific Comments:

*• What is meant by “severe” when describing the antibiotics treatment? It seems that this could mean lengthening T or changing gamma or p (line 111). These are quite different things in terms of the modeling effort, so I would find it clearer if they were not lumped into one term and if “severe” was not used through the paper but rather “duration, probability, efficacy” or other similarly informative terms.*

Reply: It means changing one of p, T, or γ. While those are different parameters, in terms of triggering the transition, they play the same role. It is, we think, also an interesting findings. We add more explanations to clarify this point.

*• Line 100-105: Setting the killing rate to gamma=1—is this reasonable, what would happen as your vary gamma?*

Reply: The effect of changing γ value is qualitatively the same with changing p or T. To show that, we added another figure to the Supplement (Fig. S1)

• The legend for Figure 1 says “The local peak of the fitness is formed at p~0.2 and the fitness value at the peak exceeds its value at 1/lambda -> infinity”. This does not look true at p~0.2 but does look true as p increases. Please clarify the legend.

Reply: Thank you for pointing out. Indeed, the last two sentences for explanation of Fig.1 (b) were redundant and the sentence that have been pointed out were the incorrect one that should have been removed. In the updated manuscript, the incorrect sentence was removed. Sorry for confusion.

• What does “fitters” on line 132 mean? More fit cells?

Reply: Yes, that was what we meant. We have replaced this word with “more fit cells”

*• The clarification of FI (line 126) should come earlier when FI is first defined. Readers familiar with Kelly bet hedging, etc will already have found this clear, but this will help hold other reader’s understanding.*

Reply: Thank you for the suggestion. We put those sentences right after we introduce FI.

• I could not find, perhaps I am missing it, the range of lambda used in the simulations for Figure 1C, 1D---what was the jump in lag between populations, and did this matter for the simulation?

Reply: In the previous manuscript, we forgot to put the actual value of the difference of the lag time between two neighboring populations, Δλ. Thank you for notifying it. We put the value and added an extra figure showing the effect of the value of Δλ (Fig.S5)

• It is not clear what x* and xL* are in Figure 3 (from the figure legend)

Reply: It is the globally and locally optimal fraction, respectively. We added the description for them in the figure caption.

• The colormap bar is not labelled on Figure 4---is this x? Would it be easier to label (i), (ii), etc from lines 247-250 on the figure? (Figure 4?)

Reply: We labeled it and moved the figure above. Thank you so much for your useful suggestions.
